# Can Reduced Irrigation Mitigate Ozone Impacts on an Ozone-Sensitive African Wheat Variety?

**DOI:** 10.3390/plants8070220

**Published:** 2019-07-12

**Authors:** Harry Harmens, Felicity Hayes, Katrina Sharps, Alan Radbourne, Gina Mills

**Affiliations:** Centre for Ecology & Hydrology, Environment Centre Wales, Deiniol Road, Bangor, Gwynedd LL57 2UW, UK

**Keywords:** ozone pollution, irrigation, wheat, photosynthesis, stomatal conductance, chlorophyll, crop yield, 1000-grain weight, harvest index

## Abstract

Ground-level ozone (O_3_) pollution is known to adversely affect the production of O_3_-sensitive crops such as wheat. The magnitude of impact is dependent on the accumulated stomatal flux of O_3_ into the leaves. In well-irrigated plants, the leaf pores (stomata) tend to be wide open, which stimulates the stomatal flux and therefore the adverse impact of O_3_ on yield. To test whether reduced irrigation might mitigate O_3_ impacts on flag leaf photosynthesis and yield parameters, we exposed an O_3_-sensitive Kenyan wheat variety to peak concentrations of 30 and 80 ppb O_3_ for four weeks in solardomes and applied three irrigation regimes (well-watered, frequent deficit, and infrequent deficit irrigation) during the flowering and grain filling stage. Reduced irrigation stimulated 1000-grain weight and harvest index by 33% and 13%, respectively (when O_3_ treatments were pooled), which compensated for the O_3_-induced reductions observed in well-watered plants. Whilst full irrigation accelerated the O_3_-induced reduction in photosynthesis by a week, such an effect was not observed for the chlorophyll content index of the flag leaf. Further studies under field conditions are required to test whether reduced irrigation can be applied as a management tool to mitigate adverse impacts of O_3_ on wheat yield.

## 1. Introduction

Tropospheric ozone (O_3_) is a secondary pollutant formed in the atmosphere by chemical reactions between the O_3_ precursors carbon monoxide, nitrogen oxides, methane, and non-methane volatile organic compounds in the presence of solar radiation [1,2]. Since the industrial revolution, concentrations over much of the Earth’s land surface have more than doubled due to anthropogenic emissions from vehicles, industry, and agriculture [1,2,3,4,5]. Since 2000, concentrations have started to decline in the eastern United States and parts of Europe due to precursor emission controls. However, O_3_ concentrations have been increasing rapidly in developing regions such as south and east Asia [6,7] and are predicted to continue to increase in the coming decades unless more stringent air pollution and climate change controls are implemented in those regions [8]. Global modelling simulation and satellite data also reveal increases in the last four decades over central Africa [7]. During biomass burning season, surface O_3_ concentrations can reach peaks up to 70 ppb in Rwanda [9] and 80 ppb in South Africa [10].

O_3_ is absorbed into leaves via the stomatal pores and is a powerful oxidant. Once inside the leaves, it reacts with biomolecules to form reactive oxygen species (ROS), triggering various cellular responses [11]. Ascorbate/ascorbic acid is thought to play a central role in scavenging ROS and signal transduction pathways, but the exact mechanism is still not fully understood [12]. Cellular and signalling responses to O_3_ vary with concentration, differ for chronic and acute O_3_ exposure, and vary among species [11]. Once defence mechanisms are overwhelmed, this can ultimately lead to programmed cell death and a reduced extent and duration of functional green leaf area, producing less photosynthate for seed fill [11]. O_3_ pollution reduces the yield of many crops, including the staple crops wheat, rice, and soybean [13,14,15], and therefore contributes to the yield gap [16]. Recently, it was estimated that O_3_ reduces the annual global yield of soybean (12.4%), wheat (7.1%), maize (6.1%), and rice (4.4%), adding up to 227 Tg of lost yield [16]. Areas at high risk of yield losses due to O_3_ are often also at risk of high losses from other biotic and abiotic stresses [16].

Producing adequate food to meet future global demand is a major challenge [17,18,19]. There is a need to develop crop cultivars or varieties that have both high productivity in future climates and high tolerance of biotic and abiotic stresses [16,19,20]. O_3_ is currently not one of the abiotic stresses included in crop breeding programmes [11,16,21], but crop ideotypes with tolerance of multiple stresses including O_3_ have been described to highlight how O_3_ effects should be included [16]. Sub-Saharan Africa (SSA) is the region at greatest food security risk in the future because, by 2050, its population will increase 2.5-fold, and demand for cereals will approximately triple [19].

Whilst improved irrigation is expected to improve crop yield in water-limited regions, it will also stimulate the stomatal uptake of O_3_ in those regions, as the leaf pores tend to be wide open in well-irrigated plants, resulting in enhanced yield loss due to O_3_ pollution [22]. For example, expanding irrigation in India and China will increase wheat yield losses due to O_3_ in major wheat growing areas of these countries at current O_3_ levels, with effects potentially even higher as O_3_ concentrations rise in coming decades [22]. The stomatal uptake of O_3_ (or Phytotoxic O_3_ Dose) is highest when soil moisture, climate conditions such as light intensity, vapour pressure deficit, and temperature are not limiting the aperture of the stomata [23,24,25]. The challenge will be to manage the amount of irrigation sustainably such that drought stress can be alleviated without significant enhancement of O_3_ stress [16]. In rice-growing areas, alternate wetting and drying irrigation (AWD) has been implemented in an attempt to reduce water usage and methane emissions [26,27]. AWD with moderate water stress increased the growth and yield of rice and reduced stomatal conductance (g_s_) compared to continuously flooded crops [28]. Reduced g_s_ will lead to a reduced O_3_ uptake, hence AWD might also be beneficial for mitigating the adverse impacts of O_3_ on rice yield. Similarly, reduced irrigation of wheat either stimulated both yield and crop water use efficiency (WUE) [29,30,31] or stimulated WUE without a yield penalty [32,33].

Other methods applied to enhance the sustainable use of water in agriculture include deficit irrigation (DI) or partial root-zone drying (PRD) [34,35]. With DI, the entire root zone receives less water than the potential evapo-transpiration. With PRD, only part of the root zone is irrigated, and the other part is left to dry to a predetermined level before the next irrigation. PRD enhances WUE of plants, potentially due to increased root–shoot surface ratio and the root hydraulic conductivity [34]. With PDR, the induction of ABA-based root-to-shoot chemical signalling might be responsible for the increase in WUE [36,37,38,39,40]. However, ABA-induced stomatal closure may not always lead to increased WUE at the whole plant level, hence growers must adapt their irrigation scheduling according to crop requirements [41]. Determining the cause of enhanced crop yields under deficit irrigation (AWD, DI or PRD) remains challenging [42].

The aim of the current study was to test whether reduced irrigation (i.e., DI) can potentially be used as a management tool to reduce the adverse impact of O_3_ on wheat. We hypothesised that reduced irrigation will reduce g_s_ and therefore the accumulated uptake of O_3_, resulting in a delay in an O_3_-induced decline in flag leaf photosynthesis and an improved yield compared to full irrigation. An O_3_-sensitive African variety of wheat [43] was exposed to low (daylight mean O_3_ concentration of 25 ppb, with peak concentrations aimed at 30 ppb) and high O_3_ (daylight mean O_3_ concentration of 45 ppb, with peak concentrations aimed at 80 ppb) for five days a week in hemi-spherical glasshouses for four weeks during flowering and grain fill. The high O_3_ treatment represents surface peak O_3_ concentrations observed in South Africa during periods of biomass burning [10]. Plants were either well-watered every day or received a reduced amount of water, either reduced every day (frequent deficit) or much reduced water every other day and well-watered the other day (infrequent deficit). Gas exchange and chlorophyll content index of the flag leaf were monitored weekly, and yield parameters were determined on maturity. In this study, reduced irrigation delayed the O_3_-induced reduction of photosynthesis of the flag leaf. Reduced irrigation did not significantly affect grain yield. However, it stimulated 1000-grain weight and harvest index, which compensated for the O_3_-induced reductions in 1000-grain weight and harvest index observed in well-watered plants. Potentially, reduced irrigation can be used as crop management tool to reduce the adverse impacts of O_3_ on wheat yield parameters. However, application and further validation is required under field conditions.

## 2. Results

### 2.1. Soil Moisture

**Figure 1:**
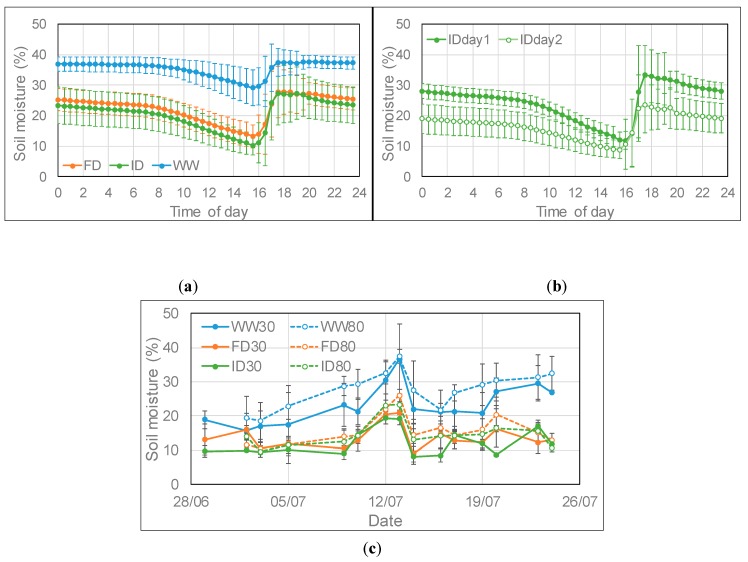
Average (± SD) daily profile of soil moisture content at low ozone (O_3_) (**a**) at three irrigation regimes (n = 27 (days)), (**b**) at ID irrigation on day 1 (full watering; n = 13 (days)) and day 2 (very limited watering; n = 14 (days)) and (**c**) average (± SD; n = 4) soil moisture content at low and high O_3_ between 14:30 and 15:00 at three watering regimes for the period 26 June–23 July 2018, with high O_3_ exposure starting on 28 June. Wheat plants were watered between 15:30 and 17:30; WW = well-watered; FD = frequent deficit irrigation; ID = infrequent deficit irrigation; 30 = peak O_3_ concentration of 30 ppb; 80 = peak O_3_ concentration of 80 ppb.

After watering in the well-watered (WW) treatment at low O_3_, the soil moisture remained rather constant throughout the evening and night and then declined steadily during the day from 8:00 onward until the following watering event (Figure 1a). Field capacity was reached at a soil moisture of about 38% as indicated by the saturation level in WW plants, a level at which water was starting to drain from the bottom of the containers. In the reduced watering treatments at low O_3_, the soil moisture declined slightly during the evening and night and then on average declined linearly at a higher rate than in the WW treatment (Figure 1a). The average decline in the WW, frequent deficit (FD) and infrequent deficit (ID) irrigation regimes was 0.97%, 1.27% and 1.39% per hour respectively between 8:00 and 15:30; for the ID irrigation the average decline was 1.75% on day 1 and 1.05% per hour on day 2 after full watering (Figure 1b). Between 14:30 and 15:00, the average measured soil moisture of the FD and ID treatment for the combined O_3_ treatments was 59% and 54% respectively of the WW treatment (Figure 1c), with respectively 29% and 20% less water being supplied than in the WW treatment. The first two weeks of the irrigation period were marked by unusually warm weather, hence some watering was required on the second day after full watering in the ID treatment to prevent drought stress occurring. From 11–13 July, it was rather cool, hence much less watering was required and a peak in soil moisture content was observed (Figure 1c). On average, the soil moisture was higher in high than low O_3_ containers due to the development of visible leaf damage and early senescence at high O_3_ (as indicated below by the early decline in chlorophyll content of the flag leaf at high compared to low O_3_). At mid-afternoon, the soil moisture content was on average 17%, 14%, and 22% higher at high compared to low O_3_ for the WW, FD, and ID treatment, respectively (Figure 1c), with less watering being required at high O_3_ as leaf injury started to develop. On average during the experimental period, 7%, 5%, and 9% less water was supplied to WW, FD, and ID containers, respectively, at high compared to low O_3_.

### 2.2. Stomatal Conductance (g_s_) of the Flag Leaf

**Figure 2:**
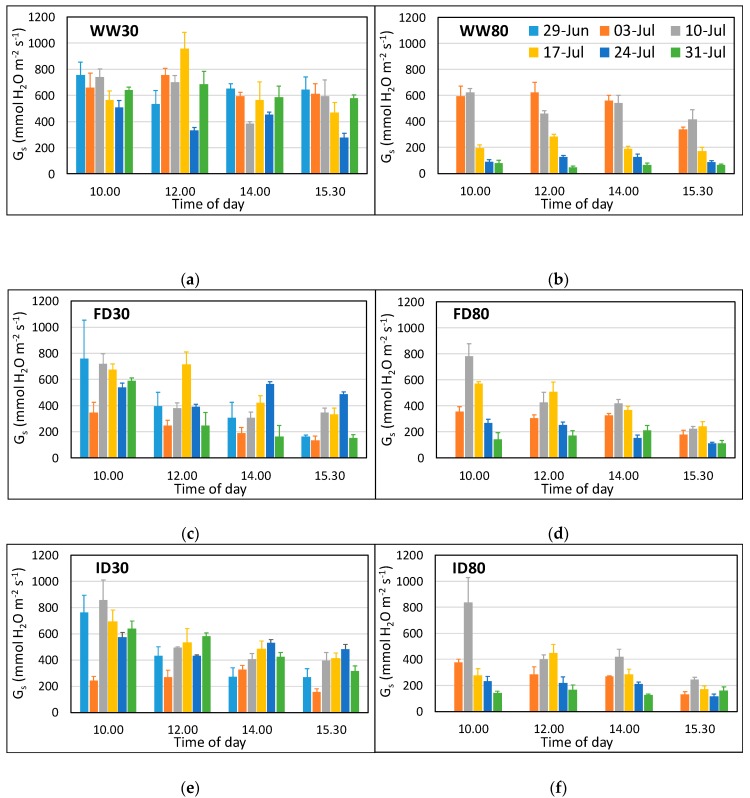
Stomatal conductance (g_s_) of the flag leaf wheat at low (**a**,**c**,**e**) and high (**b**,**d**,**f**) O_3_ concentrations and three irrigation regimes between 29 June (only measured at low O_3_) and 31 July at four different times of the day. The treatments were applied for the period 26 June–23 July 2018, with high O_3_ exposure starting on 28 June. Data for 29 June were not included in the statistical analysis as they were only obtained for low O_3_. WW = well-watered (**a**,**b**); FD = frequent deficit irrigation (**c**,**d**); ID = infrequent deficit irrigation (**e**,**f**), with low volume applied on the day before 29 June, 3 July, and 24 July; 30 = peak O_3_ concentration of 30 ppb; 80 = peak O_3_ concentration of 80 ppb. Values are averages (± SE; n = 4).

Stomatal conductance (g_s_) of the flag leaf measured using a porometer showed a significant 4-way interaction (p < 0.001) between O_3_, irrigation regime, time, and time of day (Figure 2). High O_3_ resulted in a decrease in g_s_, particularly with increasing time of exposure (strong O_3_ * time effect, p < 0.0001), reflecting the fact that impacts are determined by the accumulated stomatal flux of O_3_. The low O_3_ well-watered plants showed some fluctuation for g_s_ over time, but there was no difference between g_s_ values for 3 and 31 July, at any time of the day (Figure 2a, Table S1a). Overall, they maintained a high g_s_ throughout, with the lowest values observed on 24 July due to cloudy conditions (average photosynthetic photon flux density (PAR) was 354 µmol m^−2^ s^−1^ and average leaf temperature was 21 °C, with PAR and leaf temperature being above 1200 µmol m^−2^ s^−1^ and 25 °C respectively on other days). In contrast, in the high O_3_ well-watered treatment, g_s_ declined sharply from the third week of measurements onwards, at all times of day, reflecting adverse O_3_ impacts (Figure 2b, Table S1a). FD and ID plants at low O_3_ showed increases between 10th and 24th July, and then (depending on the time of day), declining again by 31 July. In the low O_3_ ID treatment, g_s_ remained high on 31 July for the morning measurements (Figure 2e). FD and ID plants behaved similarly at high O_3_, with g_s_ first increasing with time, reflecting the change in soil moisture conditions, and then decreasing again (to values less than those on 3 July), reflecting the onset of early senescence (Figure 2d,f, Table S1b,c). Overall, g_s_ declined during the day in plants with reduced irrigation (FD and ID; Table S2b,c).

### 2.3. Light-Saturated Photosynthesis (A_sat_) and Chlorophyll Content Index (CCI) of the Flag Leaf

**Figure 3:**
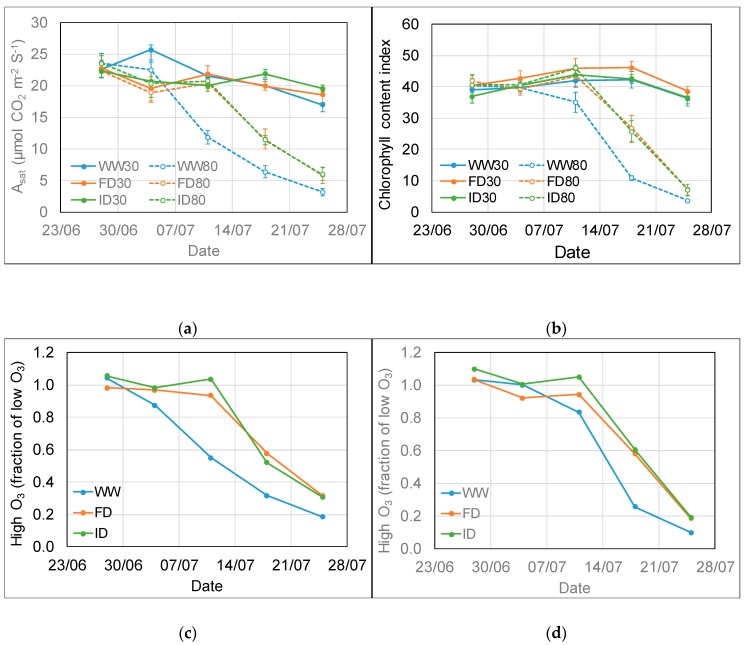
Light-saturated rate of photosynthesis (**a**,**c**) and chlorophyll content index (**b**,**d**) of the flag leaf of wheat at low and high O_3_ concentrations and three irrigation regimes between 28 June and 25 July. The bottom graphs (**c**—Asat, **d**—CCI) show the fraction of the values for both parameters at high compared to low O_3_. The treatments were applied for the period 26 June–23 July 2018, with high O_3_ exposure starting on 28 June. WW = well-watered; FD = frequent deficit irrigation; ID = infrequent deficit irrigation (with low volume applied on the day before 11 and 18 July); 30 = peak O_3_ concentration of 30 ppb; 80 = peak O_3_ concentration of 80 ppb. Values are averages (± SE; n = 4).

Whilst the light-saturated rate of photosynthesis (A_sat_) and chlorophyll content index (CCI) of the flag leaf were maintained at low O_3_ throughout the experimental period (with minor differences between irrigation treatments), both parameters started to decline after a short lag time at high O_3_ (Figure 3). The statistical analysis showed a significant 3-way interaction (p < 0.001) between O_3_ level, irrigation regime, and time for both parameters. For A_sat_, a sharp decline was first observed in WW plants between 4 and 11 July, i.e., between 6 and 13 days after starting the high O_3_ exposure, with a sharp decline at reduced irrigation occurring a week later. For CCI, plants in all irrigation treatments showed a sharp decline between 11 and 18 July, i.e., between 13 and 20 days after starting the high O_3_ exposure. On 11 July at high O_3_, A_sat_ had declined more (45%) than CCI (17%) in WW plants compared to low O_3_. On 18 July, both A_sat_ and CCI had declined by approximately 70% at high compared to low O_3_ in WW plants; at reduced irrigation, both parameters had declined by approximately 40% at high compared to low O_3_. The decline in both parameters indicated the onset of early senescence. There was no clear impact of irrigation treatment on the intrinsic (i.e., A_sat_/g_s_) or instantaneous (i.e., A_sat_/evapotranspiration) water use efficiency (WUE) of the flag leaf of wheat (data not shown).

**Figure 4:**
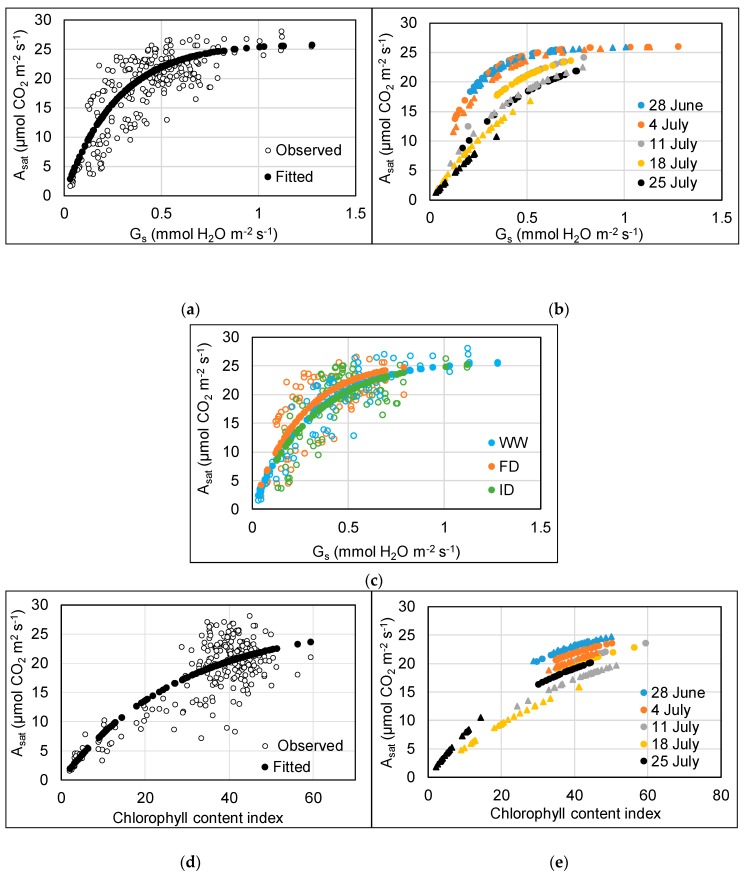
Relationship between A_sat_ and g_s_ (**a**–**c**) and CCI (**d**,**e**) of the flag leaf of wheat between 28 June and 25 July. For plots a, c, d, filled circles show the model fitted values (for fixed effects), and empty circles show the observed data. For plots b, e, these are modelled fitted values at different dates; circles = low O_3_, triangles = high O_3_.

A_sat_ showed a significant (p < 0.001) non-linear (3-parameter asymptotic exponential) relationship with both g_s_ (y = 26 – 26e^−3.4x^) and CCI (y = 27 – 27e^−0.035x^) (Figure 4). A_sat_ increased with increasing g_s_ and CCI, for the former up till approximately 0.75 mmol H_2_O m^−2^ s^−1^, after which no further increase of A_sat_ was observed (Figure 4a,d). The optimal models explained 90% (g_s_) and 81% (CCI) of the total variation in A_sat_ and contained the additional fixed effect of an O_3_ and time interaction. The rate constant (c) gradually decreased with time for both the g_s_ and CCI models (Figure 4b,e). A_sat_ also increased at a quicker rate with increasing g_s_ at low compared to high O_3_ between 11 and 25 July (p < 0.001), and the drop in rate was more evident for the high O_3_ treatment (Figure 4b). Similarly, A_sat_ increased with increasing CCI at a faster rate at low O_3_ compared to high O_3_ between 11 and 18 July (p = 0.001). For 25 July, however, there was no difference in the rate of change between low and high O_3_ in the CCI model (p = 0.50). These differences between low and high O_3_ exposure in later weeks likely reflect the detrimental impact of the accumulated O_3_ uptake on functioning of the stomata and the photosynthetic machinery, inducing early senescence.

The A_sat_ vs. g_s_ rate constant was greater for the FD than the WW (p = 0.02) and ID treatment (p < 0.01) (Figure 4c). There was no effect of water treatment on the relationship between A_sat_ and CCI (p = 0.30). The sometimes relatively high CCI compared to A_sat_ at a CCI above 26 (Figure 4d) reflects the earlier decline in A_sat_ compared to CCI in the WW treatments at 80 ppb O_3_. At low O_3,_ there were no CCI values below 30.

### 2.4. Yield and Harvest Index

**Figure 5:**
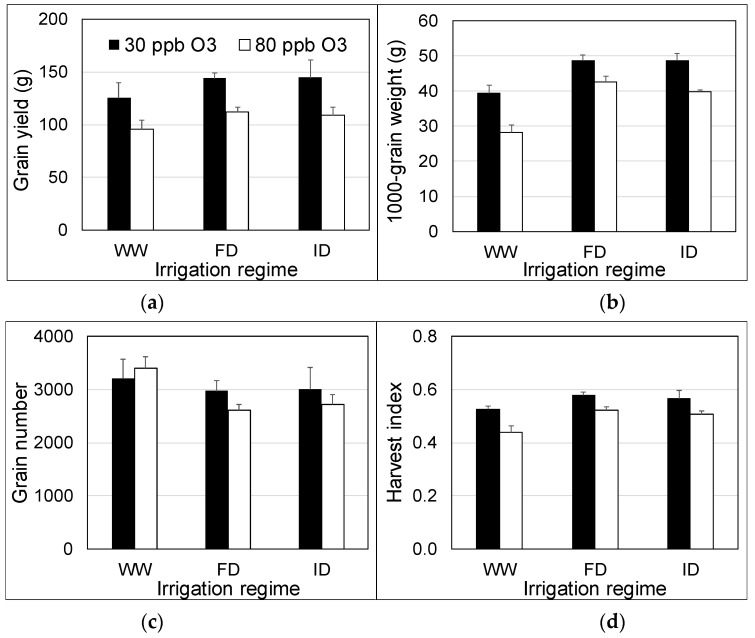
Yield ((**a**) grain yield, (**b**) 1000-grain weight, (**c**) grain number), and harvest index (**d**) of wheat at 30 and 80 ppb peak O_3_ concentrations and three irrigation regimes applied between 26 June and 23 July 2018. WW = well-watered; FD = frequent deficit irrigation; ID = infrequent deficit irrigation. Values are averages (±SE; n = 4).

High O_3_ significantly (p = 0.001) reduced grain yield by 24% on average. However, grain yield was not significantly (p = 0.27) affected by the irrigation regime, and there was no significant (p = 0.90) interaction between O_3_ concentration and irrigation regime (Figure 5a). On the other hand, 1000-grain weight was significantly (p < 0.001) reduced (20%) by high O_3_ and stimulated (33%) by reduced irrigation; there was no significant (p = 0.38) interaction between O_3_ concentration and irrigation regime (Figure 5b). The average number of grains was not significantly affected by treatments. Although the average grain number was lower for the reduced irrigation treatments, the effect of irrigation was not significant (p = 0.11) due to considerable variation between replicates (Figure 5c). Whereas high O_3_ significantly (p < 0.001) reduced (12%) the harvest index (Figure 5d), reduced irrigation significantly (p = 0.006) stimulated (13%) the harvest index. For the harvest index, there was no significant difference between FD and ID (p = 0.70), but a significant difference between WW and FD (p < 0.001) and WW and ID (p = 0.02).

## 3. Discussion

To our knowledge, this study demonstrates for the first time that deficit irrigation potentially can be applied as a management tool to mitigate adverse impacts of O_3_ on crop yield. High O_3_ significantly reduced grain yield, 1000-grain weight and harvest index but not grain number, as recently reported too for wheat in a meta-analysis from 33 experiments assessing the impact of current ambient vs. preindustrial O_3_ levels [44]. Deficit irrigation (both FD and ID) enhanced 1000-grain weight and the harvest index. However, irrigation did not significantly affect grain yield, most like due to a tendency (p = 0.11) to reduce grain number. Results therefore suggest that deficit irrigation has the potential to compensate for the adverse impact of O_3_ on 1000-grain weight and harvest index observed at full irrigation.

The results of our study support the concept that O_3_ flux represents a more biologically relevant metric of O_3_ exposure than ambient O_3_ concentration and that O_3_ flux should be the preferred metric of exposure in O_3_ effect model functions [45]. Reduced irrigation resulted in a reduction of g_s_ of the flag leaf driven by a reduction in soil moisture content and therefore a reduction in O_3_ uptake. A delay in adverse impacts of high O_3_ on flag leaf photosynthesis (A_sat_) implies that the accumulated stomatal uptake of O_3_ was lower at reduced compared to full irrigation. As the current experiment was conducted only at two levels of O_3_ exposure and over a short period, it was not feasible to develop robust O_3_ flux-effect relationships for yield parameters. Further research at a range of O_3_ concentrations during the whole growing season is needed to develop robust O_3_ flux-yield relationship and assess whether mitigation of adverse impacts of O_3_ on wheat yield at reduced irrigation can simply be explained by a reduction in the phytotoxic O_3_ dose [46]. Developing such a flux–yield relationship for the Kenyan wheat variety Korongo, a very O_3_-sensitive variety [43], would also allow comparison of the O_3_-sensitivity of an African wheat variety to those from other continents. Preferably, further research with Korongo should be conducted under Kenyan growth conditions as Korongo might perform differently under local conditions compared to the environmental conditions at the UK study site. However, currently, we are not aware of the existence of sophisticated O_3_ exposure facilities in Kenya.

The interactions between impacts of O_3_ level, irrigation regime, and time for flag leaf parameters such as g_s_, A_sat_, and CCI can be explained by the fact that adverse impacts of O_3_ are determined by the accumulated leaf uptake of O_3_ and only become apparent after a threshold has been reached. At the start of high O_3_ exposure, leaf g_s_ was highest in WW plants, resulting in the threshold for O_3_ impacts being reached first, hence an earlier decline (by about a week) in g_s_ and A_sat_ was observed in WW compared to DI plants. By the end of the high O_3_ exposure period, flag leaves in all irrigation treatments were significantly damaged and at various stage of senescence, resulting in different physiological responses to treatments compared to the healthy leaves at the start of the O_3_ exposure period. Previous research indicated that accelerated senescence might be the dominant O_3_ effect influencing wheat yield with O_3_ effects on photosynthesis only observed alongside O_3_-induced leaf senescence, monitored as CCI [47]. However, the earlier significant decline in A_sat_ compared to CCI in WW plants in the current study (resulting in a sometimes relatively high CCI compared to A_sat_ at a CCI above 26 (Figure 4d)) suggests that a decline in photosynthesis might already occur before the onset of O_3_-induced leaf senescence. Therefore, an instantaneous effect of O_3_ on photosynthesis through a reduction in the carboxylation capacity of rubisco above a critical rate of O_3_ uptake cannot be ruled out [48,49].

There is also a need to further investigate the role and mechanism of stomatal control at reduced irrigation and high O_3_ exposure on crop growth and yield. Whilst ABA appears to play a central role in root-to-shoot chemical signalling to regulate growth and water use at reduced irrigation [36,37,38,39,40], elevated O_3_ might reduce the sensitivity of stomatal closure to ABA in the presence of O_3_-induced emissions of ethylene [50,51,52]. Incomplete understanding of the physiological mechanisms driving O_3_-induced yield reduction hamper integration of O_3_ effects in crop modelling [47,53,54]. It is not fully understood yet which processes affected by O_3_ uptake at the leaf level are most important in driving ultimate yield loss. In crop modelling, there is a need to use a dynamic approach [47,55] and to improve the understanding of leaf-area dynamics in response to O_3_ [54].

There was no clear impact of irrigation treatment on the intrinsic (i.e., A_sat_/g_s_) or instantaneous (i.e., A_sat_/evapotranspiration) water use efficiency (WUE) of the flag leaf of wheat (data not shown) as again there was a significant interaction with time (i.e., date of measurement). WUE of the flag leaf was generally quite stable in WW plants, but variation in flag leaf WUE in reduced irrigation plants meant that in some weeks their WUE was higher than in WW plants, whereas the opposite was true for other weeks. It should be noted, however, that it is difficult to upscale impacts of treatments on WUE of the flag leaf to whole plant WUE and consequences for crop yield without detailed information on other important variables such as leaf position (affecting light environment) and dark respiration [56].

Although the current study did not show a significant impact of DI on grain yield (but a significant stimulation of 1000-grain weight and harvest index), some field studies have shown a positive impact of DI on wheat yield. Maximum wheat yield and a high crop WUE was achieved using drip irrigation to maintain a soil moisture content of 60% of field capacity in the North China Plain [29]. Another study reported maximum wheat yield and irrigation WUE for the North China Plain at a relative soil water content of 75% [30]. High-level irrigation made wheat produce tillers for a longer period, delaying the time it reaches maturity, resulting in an increase in biomass allocation to straw [29] and a reduction in biomass allocation to grains [29,30]. Whilst full irrigation showed a tendency to reduce grain weight and stimulate straw weight in our study (data not shown), a significant difference (p ≤ 0.05) in straw weight was only found between WW and FD plants (when O_3_ treatments were pooled; O_3_ did not affect straw weight). Under semi-arid environmental conditions in Pakistan, 20% DI in combination with raised bed cultivation and mulching improved wheat yield [31]. Other field studies have shown that wheat yield [32,33] was not significantly affected at 25–30% DI compared to full irrigation. However, the crop WUE was much higher at DI, and water saving could be achieved without a yield penalty. There is a clear need to repeat the current study under field conditions where the dynamics of the soil water content will be different from container studies, as the soil water content declines quickly in containers on a daily basis, and daily watering is required to prevent drought stress. When conducting such field studies, we recommend exposing wheat to a range of elevated O_3_ concentrations throughout the growing season—for example, using free air O_3_ exposure systems, with DI applied at different stages of development. Previous studies have shown that the impact of DI on wheat yield and quality varies with the developmental stage of wheat [57,58].

Such field studies should test the hypothesis that reduced irrigation will mitigate adverse impacts of O_3_ on wheat yield in areas with high ambient O_3_ concentrations, such as large regions in South and East Asia, including China and India [16,22,55]. Whilst a more sustainable water use in irrigated crops through reduced irrigation provides a feasible crop management solution to reduce the yield gap in crops, there is a need as well to improve the resilience of crops to environmental stresses such as drought, heat, pest and diseases, and O_3_ pollution in the long term through crop breeding programmes [20,22,59]. It might not be feasible to meet future SSA cereal demand on existing production area by decreasing the yield gap alone. However, any attempts to promote the sustainable expansion of irrigated production areas [19] should bear in mind that rising O_3_ concentrations in SSA [7] are likely to reduce the yield potential when soil moisture becomes less of a limiting factor in the stomatal uptake of O_3_ in (semi-)arid areas.

## 4. Materials and Methods

### 4.1. Plant Material, Experimental Site and Treatments

The experiment was conducted in 2018 at the Centre for Ecology & Hydrology (CEH) air pollution facility at Abergwyngregyn, North Wales (53.2°N, 4.0°W). On 10 May, seeds of the Kenyan wheat (*Triticum aestivum* L.) variety ‘Korongo’ (Kenya Agricultural Research Institute, Njoro, Kenya) were sown in 24 containers (0.3 m × 0.3 m × 0.3 m) filled to 25 litres with John Innes No. 3 compost (J. Arthur Bowers, LBS Horticulture, Colne, UK). Korongo is a high-yielding Kenyan wheat variety (yield potential of 8.5 tonnes ha^−1^) and is outstanding for baking and confectionery qualities [60]. Seeds were sown in four rows 7 cm apart, resulting in a seedling density of approximately 330 seedlings per m^2^. The containers were randomly distributed in one hemispherical glasshouse (solardomes; 3 m diameter, 2.1 m height) at a low O_3_ profile with weekly maximum peaks up till 31 ppb and a daylight mean O_3_ concentration of 19 ppb (Table 1) until 26 June. Climatic conditions in the solardome fluctuated with the outside ambient climatic conditions; a summary of the climatic conditions is provided in Table 1. The containers were well-watered to maintain soil moisture near field capacity.

**Figure 6:**
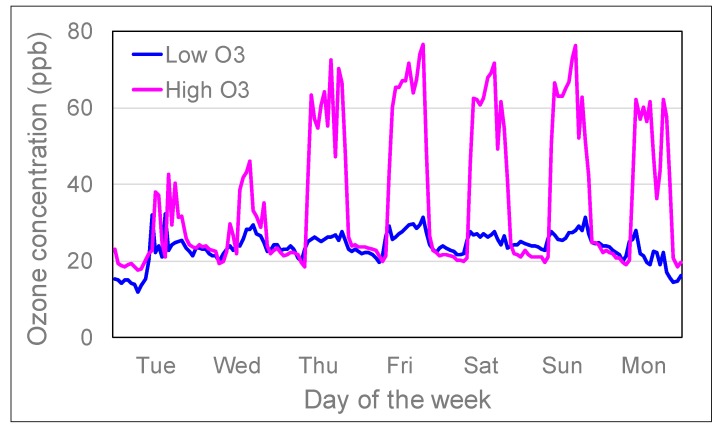
Average hourly O_3_ concentrations in ‘low’ and ‘high’ solardomes at Abergwyngregyn, near Bangor, North Wales, between 26 June and 23 July in 2018.

On Tuesday 26 June at approximately mid-anthesis stage, randomly half of the containers were transferred to one solardome at a high O_3_ profile for four weeks with weekly maximum peaks up till 93 ppb (Table 1) and an average hourly O_3_ concentration up to 76 ppb (Figure 6). The other half of the containers remained in the solardome with the low O_3_ profile. This period represents the most O_3_-sensitive developmental stage of wheat [14]. The wheat was exposed to peak O_3_ concentrations five consecutive days a week (Thu–Mon) between 8 am and 8 pm (starting two days after transfer on 28 June, representing an O_3_ episode at the high O_3_ exposure) with lower peaks during the other two days (Tue–Wed). During ‘night’ time average hourly O_3_ concentrations were kept between 18–25 ppb, peaking at 46 ppb during daytime on the other two days of the week (Figure 6). At low O_3_, the average hourly concentration reached values up to 32 ppb between 26 June and 23 July. During the four weeks that wheat was exposed to low and high O_3_, three irrigation regimes were applied (four replicate containers per irrigation regime per O_3_ concentration): well-watered every day (WW: well-watered), reduced watering every day (FD; frequent deficit), well-watered every other day with limited watering on the other day to prevent drought stress (ID: infrequent deficit). For the remainder of the experiment, all containers were kept in the low O_3_ solardome again and well-watered until harvest. Hence, apart from during the four weeks of different irrigation regimes and high O_3_ treatment, all plants were grown in the same solardome. As previous assessments had shown that climatic conditions do not vary significantly between the solardomes [61] and in order not to disturb the plants too much during the four weeks, it was decided not to swap the O_3_ treatments and plants between the two solardomes.

The solardomes were ventilated at a rate of two air changes per minute, and charcoal-filtered air was injected with controlled amounts of O_3_. O_3_ was provided by a G11 O_3_ generator (Ozone Industries, Andover, UK) equipped with a Sequal 10 oxygen concentrator (Pure O2, Manchester, UK). Concentrations were determined by a computer-controlled O_3_ injection system (Lab VIEW version 2012, National Instruments, Austin, Texas, US). O_3_ was distributed to each solardome via PTFE tubing, with the concentration inside each solardome measured for 5 min every 30 min using two O_3_ analyzers (400a, Enviro Technology Services, Stroud, UK; 49i, Thermo Fisher Scientific, Franklin, Massachusetts, USA) of matched calibration. In one solardome, ambient air temperature, photosynthetically active radiation (PAR), temperature and relative humidity were continuously monitored by an automatic weather station (Skye Instruments Ltd., Llandrindod Wells, UK) and recorded every five minutes.

### 4.2. Watering and Soil Moisture Measurements

The containers were watered daily with different volumes of water during the period of differential irrigation and exposure to low and high O_3_ concentrations. The soil moisture was monitored on weekdays between 14:30–15:00 pm in the top 6 cm near the middle of the container with a handheld soil moisture sensor (Theta Probe ML3, AT Delta-T Devices Ltd., Burwell, UK). Depending on measured soil moisture and predicted weather conditions for the next day, the volume of water to be added per container between 15:30–17:30 was determined. The aim of the WW treatment was to maintain soil moisture above 20%, the aim of the reduced irrigation treatments was not to drop soil moisture below 10%, with ID being in the range of 10–15% 2 days after being well-watered and FD being around 15% when monitoring soil moisture. In the low O_3_ treatment, permanent soil moisture sensors of the same make were installed in two containers of each watering regime, and the soil moisture was logged (GP2 Data Logger, AT Delta-T Devices Ltd., Burwell, UK) every half an hour to obtain daily profiles of soil moisture changes.

### 4.3. Leaf Gas Exchange and Chlorophyll Context Index

Between 29 June (low O_3_) or 3 July (high O_3_) and 31 July, stomatal conductance of the flag leaf was measured weekly under ambient conditions on Tuesdays between 10.00–10.45, 12.00–12.45, 14.00–14.45, and 15.30–16.15 using a porometer (AP4 Poromoter, Delta T Devices Ltd., Burwell, UK) to determine the daily profile. The leaf temperature and ambient light levels were recorded too. At the start of the high O_3_ exposure (28th July) and weekly thereafter on Wednesdays until 25th July, the light-saturated rate of photosynthesis (A_sat_) and stomatal conductance (g_s_) of the flag leaf were measured weekly between 10.30 and 14.00 using a portable infrared gas analyser (LI-6400XT, LI-COR, Lincoln, Nebraska, USA). The mean environmental conditions in the leaf chamber were as follows: air temperature of 25 °C, CO_2_ concentration of incoming air of 400 µmol mol^−1^, light of 1500 µmol m^−2^ s^−1^ (6400 LED light source), relative humidity of outgoing air of 68% on average, leaf vapour pressure deficit leaf of 1.2 kPa on average. At the same time, the chlorophyll content index (CCI) of the flag leaf was measured using a CCM200 (ADC, UK). The CCM200 measures non-destructively the ratio of optical transmission at 931 nm to optical transmission at 653 nm waveband.

### 4.4. Wheat Yield and Harvest Index

Ripening of the ears occurred at a different rate in the different treatments, hence it was decided to harvest individual ears when they had ripened to prevent loss of ripened seeds. On average, 75% or more of the ears were harvested between 10 (FD80 treatment) and 19 August (WW30 treatment). The ears were stored at room temperature. When all the ears were harvested, straw (leaves and stems) was harvested too, dried at 60 °C and weighed. Ears were threshed (Minibatt+, Godé, France), the grains were weighed, and the harvest index was calculated as the ratio of the grain weight and ‘grain weight plus straw weight’. In addition, the weight of 100 grains per container was determined to calculate the 1000-grain weight.

### 4.5. Statistical Analyses

All statistical analyses were run using R [62]. To investigate the effect of O_3_ and irrigation treatments on g_s_, A_sat_ and CCI, the R package ‘lme4′ [63] was used to run linear mixed effects models, including the fixed effects of O_3_ level, irrigation treatment, time (categorical), where relevant time of day (categorical, only relevant for g_s_ as shown in Figure 2), and interactions between these variables. A random effect of container number was also included. For the g_s_ model, based on the porometer measurements, continuous variables for leaf temperature (quadratic term) and ambient light were also added. As CCI was generally stable for the first three weeks (irrespective of the treatments), values for 28 June, 4 and 11 July were included in one category (level 0), values for 18 July were put into a second category (level 1), and values for 25 July were put into a third category (level 2). Therefore, the ‘time’ variable in the CCI model was categorical, with 3 levels. The R package ‘emmeans’ [64] was used to investigate differences between the treatments. To determine the relationship between A_sat_ vs. g_s_ and A_sat_ vs. CCI, non-linear mixed effects models were run using the R package (‘nlme’) [65]. A 3-parameter asymptotic function of the form y = a –be ^–cx^ was used. The asymptote (a) was derived from the plot of the observed data, and b was calculated using a set intercept of 0, following ‘a – b = intercept.’ The rate constant ‘c’ was calculated as

c = −(log((a − y)/b)/x)

where ‘x,y’ are the coordinates for the point on the plot of the observed data where the curve is rising most steeply [66]. Model sets were created, including CCI or g_s_ as a fixed effect, and container number as a random effect, with additional models including time, O_3_ treatment, and/or irrigation treatment as fixed effects. The variation explained by the final models (r^2^) was calculated by comparing the residual variance with the total variation in the models.

For all mixed models (linear and non-linear), the optimal model was chosen by examination of Akaike’s Information Criterion (AIC) and the Bayesian Information Criterion (BIC). The model with the lowest AIC value is the optimal model, while models differing in <2, 4–7, >10 AIC from the top model have substantial, considerably less and no empirical support respectively [67]. The BIC provides a more conservative estimate, penalising models with many parameters [68]. Likelihood ratio tests were then used to determine p-values for the fixed effects. To investigate the effect of O_3_ and irrigation treatments on yield and harvest index, linear models with normal error including the categorical predictor O_3_ and irrigation (and their interaction) were run. For the grain weight and grain number models, number of plants per pot was included as an additional covariate as the germination rate was not 100% in all the containers. Post-hoc Tukey tests were used to investigate differences between irrigation treatments. For all linear models, residual plots were examined for uneven spread and non-normality. Data transformations were carried out if necessary, e.g., square root or log transformation.

## 5. Conclusions

Our study indicates that reduced irrigation can be applied as a management tool to partially or fully mitigate (depending on yield parameter) the adverse impacts of O_3_ on wheat yield. Reduced irrigation reduces g_s_ and therefore the stomatal uptake of O_3_, resulting in a delay in adverse impacts of O_3_ on flag leaf photosynthesis during flowering and grain filling. Hence, reduced irrigation enables wheat plants to maintain photosynthate production for longer during the grain-filling period at elevated levels of O_3_. The mechanism of the interactive impacts of reduced irrigation and O_3_ on g_s_ and subsequent wheat yield require further investigation and validation under field conditions. Field experiments should be conducted at various O_3_ concentrations to enable the development of robust O_3_ flux-effect relationships for wheat yield at various irrigation regimes.

## Figures and Tables

**Table 1:** Summary of O_3_ treatments and climate conditions in the solardomes in 2018.

Date (2018).	10/5–25/6	26/6–23/7		24/7–9/8
**Ozone exposure**	Low	Low	High	Low
- Weekly max. mean (ppb)	30.7	41.8	92.6	34.6
- 24 hr mean (ppb)	18.9	23.7	36.5	24.5
- Daylight mean (ppb)	19.3	24.8	45.0	27.6
**Air temperature**				
- 24 hr mean (°C)	20.1	24.9	24.9	22.2
- Daylight mean (°C)	24.6	31.4	31.4	25.2
- Daily max. mean (°C)	27.7	33.9	33.9	30.2
**Photosynthetically active radiation (PAR)**				
- Daylight mean (µmol m^−2^ s^−1^)	633	725	725	557
- Daily max (µmol m^−2^ s^−1^)	1033	1129	1129	979

## Supplementary Materials

The following are available online at https://www.mdpi.com/2223-7747/8/7/220/s1, Table S1: Post-hoc test results (p-values) for mixed model investigating the effect of varying water treatments (WW: well-watered; FD: frequent deficit; ID: infrequent deficit) and O_3_ treatments (low O_3_ = 30 ppb, high O_3_ = 80 ppb) on stomatal conductance (g_s_; mmol H_2_O m^−2^ s^−1^), Table S2: Post-hoc test results (p-values) for mixed model investigating the effect of varying water treatments (WW: well-watered; FD: frequent deficit; ID: infrequent deficit) and O_3_ treatments (low O_3_ = 30 ppb, high O_3_ = 80 ppb) on stomatal conductance (g_s_; mmol H_2_O m^−2^ s^−1^).
