# Supplementary material for: Can Reduced Irrigation Mitigate Ozone Impacts on an Ozone-Sensitive African Wheat Variety?
Source: Plants (Basel). 2019 Jul 12;8(7):220. doi: 10.3390/plants8070220 (PMC6681504; doi:10.3390/plants8070220)
Supplement: Supplementary file 1 [file plants-08-00220-s001.pdf]

Table S1. Post-hoc test results (p-values) for mixed model investigating the effect of varying water treatments (WW: well-watered; FD: frequent deficit; ID: infrequent deficit) and O_3 treatments (low O_3 = 30 ppb, high O_3 = 80 ppb) on stomatal conductance (g_s; mmol H_2O m^{-2} s^{-1}). The values show **differences between dates** (1 - 5 = 3rd, 10th, 17th, 24th and 31st July 2018 respectively) for each time of day. Bold p-values show statistical significance ($p \leq 0.05$).

**a)**		**Time of day**						**Time of day**			
**WW30**	**Date**	**10:00**	**12:00**	**14:00**	**15:30**	**WW80**	**Date**	**10:00**	**12:00**	**14:00**	**15:30**
	1 vs 2	0.8525	0.9827	0.1267	1.0000		1 vs 2	0.9999	0.9928	0.9975	0.9487
	1 vs 3	0.9996	0.9308	0.1105	0.8059		1 vs 3	**<0.0001**	**0.0051**	**<0.0001**	**0.0001**
	1 vs 4	0.4201	**0.0050**	0.6507	**0.0088**		1 vs 4	**<0.0001**	**<0.0001**	**<0.0001**	**<0.0001**
	1 vs 5	0.9943	0.9033	0.9981	0.999		1 vs 5	**<0.0001**	**<0.0001**	**<0.0001**	**<0.0001**
	2 vs 3	0.7436	0.6654	1.0000	0.8383		2 vs 3	**<0.0001**	**0.0204**	**<0.0001**	**<0.0001**
	2 vs 4	0.0522	**0.0282**	0.8524	**0.0109**		2 vs 4	**<0.0001**	**<0.0001**	**<0.0001**	**<0.0001**
	2 vs 5	0.9748	0.9971	0.2376	0.9977		2 vs 5	**<0.0001**	**<0.0001**	**<0.0001**	**<0.0001**
	3 vs 4	0.5517	**0.0002**	0.8239	0.1732		3 vs 4	**<0.0001**	**0.0229**	0.0951	**0.0199**
	3 vs 5	0.9731	0.4475	0.2120	0.6554		3 vs 5	**<0.0001**	**<0.0001**	**<0.0001**	**<0.0001**
	4 vs 5	0.2104	0.0709	0.8263	**0.0037**		4 vs 5	0.1361	**<0.0001**	**0.0005**	0.1564

**b)**		**Time of day**						**Time of day**			
**FD30**	**Date**	**10:00**	**12:00**	**14:00**	**15:30**	**FD80**	**Date**	**10:00**	**12:00**	**14:00**	**15:30**
	1 vs 2	**0.0041**	0.1026	**0.0331**	**<0.0001**		1 vs 2	**0.0017**	0.3928	0.5159	0.2463
	1 vs 3	**0.0012**	**<0.0001**	**0.0007**	**0.0001**		1 vs 3	0.1413	0.0795	0.8243	0.0778
	1 vs 4	**0.0312**	0.0572	**<0.0001**	**<0.0001**		1 vs 4	0.6443	0.9675	**0.0103**	0.7542
	1 vs 5	0.1367	0.9993	0.9996	0.9808		1 vs 5	**<0.0001**	**0.0244**	0.0909	0.3800
	2 vs 3	0.9970	0.0502	0.7940	0.9120		2 vs 3	0.5847	0.9314	0.9865	0.9844
	2 vs 4	0.9696	0.9993	**0.0218**	0.4217		2 vs 4	**<0.0001**	0.1144	**<0.0001**	**0.0116**
	2 vs 5	0.7436	0.0569	**0.0182**	**<0.0001**		2 vs 5	**<0.0001**	**<0.0001**	**0.0006**	**0.0014**
	3 vs 4	0.8664	0.0912	0.3111	0.0770		3 vs 4	**0.0025**	**0.0128**	**0.0002**	**0.0019**
	3 vs 5	0.5275	**<0.0001**	**0.0003**	**0.0013**		3 vs 5	**<0.0001**	**<0.0001**	**0.0038**	**0.0002**
	4 vs 5	0.9778	**0.0299**	**<0.0001**	**<0.0001**		4 vs 5	**0.0027**	0.1309	0.9400	0.9754

**c)**		**Time of day**						**Time of day**			
**ID30**	**Date**	**10:00**	**12:00**	**14:00**	**15:30**	**ID80**	**Date**	**10:00**	**12:00**	**14:00**	**15:30**
	1 vs 2	**<0.0001**	**0.0004**	0.7728	**<0.0001**		1 vs 2	**0.0011**	0.1255	0.0543	**0.0135**
	1 vs 3	**<0.0001**	**0.0009**	0.3982	**<0.0001**		1 vs 3	0.6192	0.0713	0.9410	0.7028
	1 vs 4	**<0.0001**	**0.0076**	**0.0415**	**<0.0001**		1 vs 4	0.1374	0.9010	0.9505	0.9203
	1 vs 5	**<0.0001**	**<0.0001**	0.7347	**<0.0001**		1 vs 5	**<0.0001**	**0.0448**	**0.0026**	0.9970
	2 vs 3	0.9835	0.9998	0.9751	0.9997		2 vs 3	**<0.0001**	0.9993	0.2920	0.3131
	2 vs 4	0.7082	0.9393	0.4648	0.6656		2 vs 4	**<0.0001**	**0.0109**	**0.0060**	**0.0007**
	2 vs 5	0.9138	0.9546	1.0000	0.4335		2 vs 5	**<0.0001**	**<0.0001**	**<0.0001**	**0.0378**
	3 vs 4	0.9478	0.9751	0.8298	0.7828		3 vs 4	0.8888	**0.0049**	0.5713	0.2172
	3 vs 5	0.9978	0.9050	0.9838	0.3195		3 vs 5	**0.0026**	**<0.0001**	**0.0001**	0.8823
	4 vs 5	0.9934	0.5785	0.5070	**0.0214**		4 vs 5	**0.0481**	0.3200	**0.0280**	0.7645

Table S2. Post-hoc test results (p-values) for mixed model investigating the effect of varying water treatments (WW: well-watered; FD: frequent deficit; ID: infrequent deficit) and O_3 treatments (low O_3 = 30 ppb, high O_3 = 80 ppb) on stomatal conductance (g_s; mmol H_2O m^{-2} s^{-1}). The values show **differences between times of day** for each date in 2018. Bold p-values show statistical significance ($p \leq 0.05$).

**a) WW30**					
	**3rd July**	**10th July**	**17th July**	**24th July**	**31st July**
**Time of day**					
10:00 vs 12:00	0.9780	0.7154	0.4625	0.1974	0.7950
10:00 vs 14:00	0.9871	**0.0011**	**0.0374**	0.9989	0.7292
10:00 vs 15:30	0.7616	0.1931	0.2132	**0.0239**	0.6960
12:00 vs 14:00	0.9046	0.1036	**0.0012**	0.2842	1.0000
12:00 vs 15:30	0.5988	0.8863	**0.0111**	0.9345	0.9999
14:00 vs 15:30	0.9279	0.3292	0.8905	0.0513	0.9999

**WW80**					
	**3rd July**	**10th July**	**17th July**	**24th July**	**31st July**
**Time of day**					
10:00 vs 12:00	0.7727	0.5627	0.7867	0.1743	0.1818
10:00 vs 14:00	0.9758	0.9281	0.9672	0.2719	0.9908
10:00 vs 15:30	**0.0044**	0.0662	0.2859	0.9995	0.9900
12:00 vs 14:00	0.9437	0.8900	0.5608	0.9804	0.3314
12:00 vs 15:30	0.1731	0.7970	0.0690	0.1656	0.3354
14:00 vs 15:30	**0.0257**	0.2898	0.5945	0.2631	1.0000

**b) FD30**					
	**3rd July**	**10th July**	**17th July**	**24th July**	**31st July**
**Time of day**					
10:00 vs 12:00	0.6587	0.1158	0.9984	0.5186	**0.0014**
10:00 vs 14:00	**0.0382**	**0.0053**	0.0535	0.9874	**<0.0001**
10:00 vs 15:30	**0.0001**	**0.0344**	**0.0006**	0.9826	**<0.0001**
12:00 vs 14:00	0.4166	0.6878	**0.0344**	0.3243	0.4365
12:00 vs 15:30	**0.0060**	0.9617	**0.0003**	0.7492	0.0609
14:00 vs 15:30	0.2923	0.9272	0.5219	0.8961	0.7466

**FD80**					
	**3rd July**	**10th July**	**17th July**	**24th July**	**31st July**
**Time of day**					
10:00 vs 12:00	0.9035	**0.0368**	0.9720	1.0000	0.5232
10:00 vs 14:00	0.9464	**0.0308**	0.2723	0.0696	0.1964
10:00 vs 15:30	**0.0003**	**<0.0001**	**0.0008**	**0.0006**	0.8244
12:00 vs 14:00	0.9992	0.9999	0.5194	0.0619	0.9253
12:00 vs 15:30	**0.0038**	**0.0096**	**0.0040**	**0.0005**	0.1190
14:00 vs 15:30	**0.0024**	**0.0117**	0.1705	0.4494	**0.0245**

c) ID30					
	**3rd July**	**10th July**	**17th July**	**24th July**	**31st July**
**Time of day**					
10:00 vs 12:00	0.9996	0.1599	0.3148	0.3896	0.9524
10:00 vs 14:00	0.2536	**0.0055**	0.1286	0.9783	0.0781
10:00 vs 15:30	**0.0013**	**0.0024**	**0.0256**	0.7774	**0.0001**
12:00 vs 14:00	0.3047	0.6028	0.9657	0.6377	0.2391
12:00 vs 15:30	**0.0008**	0.4527	0.6834	0.9213	**0.0007**
14:00 vs 15:30	**<0.0001**	0.9952	0.9186	0.9463	0.1875

ID80					
	**3rd July**	**10th July**	**17th July**	**24th July**	**31st July**
**Time of day**					
10:00 vs 12:00	0.3276	0.0078	0.0942	0.9945	0.9285
10:00 vs 14:00	0.1987	**0.0094**	0.9972	0.9828	0.9429
10:00 vs 15:30	**<0.0001**	**<0.0001**	0.0814	**0.0023**	0.9847
12:00 vs 14:00	0.9921	0.9999	0.1462	0.9994	0.6436
12:00 vs 15:30	**0.0045**	0.0521	**<0.0001**	**0.0054**	0.9940
14:00 vs 15:30	**0.0112**	**0.0446**	**0.0497**	**0.0080**	0.7955
